# Study on the Thermal Conductivity Characteristics for Ultra-Thin Body FD SOI MOSFETs Based on Phonon Scattering Mechanisms

**DOI:** 10.3390/ma12162601

**Published:** 2019-08-15

**Authors:** Guohe Zhang, Junhua Lai, Yali Su, Binhong Li, Bo Li, Jianhui Bu, Cheng-Fu Yang

**Affiliations:** 1 School of Microelectronics, Xi’an Jiaotong University, Xi’an 710049, Shaanxi, China; 2 School of Mechanical Engineering, Xi’an Shiyou University, Xi’an 710065, Shaanxi, China; 3 Institute of Microelectronics of Chinese Academy of Sciences, Beijing 100029, China; 4 Key Laboratory of Silicon Device Technology, Chinese Academy of Sciences, Beijing 100029, China; 5 Department of Chemical and Materials Engineering, National University of Kaohsiung, No. 700, Kaohsiung University Rd. Nan-Tzu District, Kaohsiung 811, Taiwan

**Keywords:** UTB-FD SOI MOSFET, self-heating effect, thermal conductivity, phonon scattering, heat diffusion

## Abstract

The silicon-on-insulator (SOI) metal-oxide-semiconductor field-effect transistors (MOSFETs) suffer intensive self-heating effects due to the reduced thermal conductivity of the silicon layer while the feature sizes of devices scale down to the nanometer regime. In this work, analytical models of thermal conductivity considering the self-heating effect (SHE) in ultra-thin body fully depleted (UTB-FD) SOI MOSFETs are presented to investigate the influences of impurity, free and bound electrons, and boundary reflection effects on heat diffusion mechanisms. The thermal conductivities of thin silicon films with different parameters, including temperature, depth, thickness and doping concentration, are discussed in detail. The results show that the thermal dissipation associated with the impurity, the free and bound electrons, and especially the boundary reflection effects varying with position due to phonon scattering, greatly suppressed the heat loss ability of the nanoscale ultra-thin silicon film. The predictive power of the thermal conductivity model is enhanced for devices with sub-10-nm thickness and a heavily doped silicon layer while considering the boundary scattering contribution. The absence of the impurity, the electron or the boundary scattering leads to the unreliability in the model prediction with a small coefficient of determination.

## 1. Introduction

Ultra-thin body (UTB) fully depleted (FD) silicon-on-insulator (SOI) metal-oxide-semiconductor field-effect transistors (MOSFETs) have emerged as the viable solution to the extreme downscaling of CMOS technology to the sub-14-nm nodes, because of its dramatic suppression of short-channel effects (SCEs), and its superiority of low-power high-speed application [1,2,3]. The scaling down of the device dimension leads to a few nanometers’ thickness of silicon film above the buried-oxide (BOX) of SOI structure and high power densities, resulting in the dramatic decrease of thermal conductivity of silicon in nanoscale SOI device [4,5,6]. In such a nanoscale SOI device, the aggravated self-heating effect (SHE) is ubiquitous because of the increased heat generation and the reduced thermal diffusion [7,8]. The thermally induced unreliability caused by the SHEs is a pressing issue for advanced UTB SOI MOSFET, which is positively activated in lattice temperature [9,10].

In order to effectively evaluate and then suppress the SHE, considerable research efforts have been devoted to the electro-thermal simulation [11,12,13], measurement [14,15] and theoretical analysis and modeling [16,17,18] of SHEs, attempting to reveal the heat generation and diffusion physical mechanisms in scaled nanoscale SOI devices. However, the constant or simple temperature-dependent value of the thermal conductivity was usually adopted for modeling the SHEs in the silicon thin film with different doping concentrations, it was even dramatically influenced by the thickness, the dopant and the temperature of the material [19,20]. Thus, a precise thermal conductivity model has become essential for accurately evaluating the self-heating effects (SHEs) which is a critical concern while improving the performance and the reliability of the device [21]. In order to refine the model of the lattice thermal conductivity for a nanoscale material, the solution of the Boltzmann equation is approximated by the use of the relaxation time concept in Reference [22]. The relaxation time-based model of Holland could be used to perfectly account for the influences of the phonon scattering, the heat capacity and the phonon velocity on the heat diffusion mechanisms in the thin silicon film [23]. Among these factors, the phonon scattering plays a key role in the thermal conductivity of silicon layer in nanoscale SOI MOSFET with low doping concentrations or even an un-doped channel. Therefore, a more detailed description of phonon transport has attracted much attention for enhancing the prediction power of the thermal conductivity models. Liu et al. established a thermal conductivity model at high temperatures based on the phonon-impurity in the silicon [24]. The accuracy of the thermal conductivity model for silicon is further improved in Asheghi’s work by taking the phonon-impurity and the phonon-electron scattering into consideration [25]. The spatial characteristic of thermal conductivity cannot be accounted for by their models because the thermal conductivity degradation caused by the silicon layer boundaries is modeled by the only thickness-dependent reduction terms. In Vasileska’s work [26], the temperature and thickness dependence of thermal conductivity in silicon thin film is established based on a simple temperature-dependent material thermal conductivity, which cannot explain the influence of the dopant. In our previous work [27], the free and bound state electron and boundary scattering were introduced to modify the phonon scattering rate for the nanoscale silicon, based on Asheghi’s work. A more accurate temperature and thickness dependent thermal conductivity model is proposed. However, the influence of different factors, such as the impurity, the electron and the boundary scattering, has not been quantitatively analyzed. The reliability of the different models is still ambiguous for modeling thermal conductivity in silicon with different doped concentrations and thicknesses. Thus, the analysis of temperature, thickness and the doping dependent thermal conductivity model is essential for accurately modeling the SHEs in Nano-SOI MOSFET.

In this paper, an analytical thermal conductivity model considering the temperature, the depth, the thickness and the doping concentration in silicon film is presented to investigate the SHEs of ultra-thin body SOI MOSFETs. The thermal dissipation mechanisms are analyzed by modifying the heat capacity, the relaxation time with Debye model and the scattering rate of the phonon. The influences of the impurity, the free and bound electron and the boundary reflection effects on the heat diffusion are discussed based on the thermal conductivity model. The reliabilities of the different models are studied by introducing the coefficient of determination for the nonlinear regression.

## 2. Thermal Conductivity Model Derived with SHEs

The thermal generation and diffusion mechanisms should be considered to study the SHEs. Based on the analysis of carrier mobility, heat generation can be seen as a static process when a SOI MOSFET works in a saturation state. The thermal resistance model can be used to describe the heat transport process [28]. The function is treated as a blocking capacity to heat transmission, which is proportional to the sample’s length of conduction path (*L*) and inversely to the cross-sectional area (*S*). So that the thermal resistance can be defined as

(1) $$R_{th}=\frac{L}{\lambda S}$$

where λ is the thermal conductivity. The Fourier law of heat conduction indicates that the heat flux is proportional to the temperature gradient [29], which could be expressed as

(2) $$Q_{x}=-\lambda \frac{\partial T}{\partial x}$$

where $Q_{x}$ is the heat flux per unit time vertically through unit area. The heat flux generated in the silicon layer dissipates through silicon film by the collisions between ions and the phonon movement and can be defined as

(3) $$Q_{x}=-\frac{1}{3}C_{V}{\bar{\upsilon }}^{2}\tau \frac{dT}{dx}$$

where $C_{V}$ is the phonon specific heat per unit volume. $\bar{\upsilon }$ is the average phonon velocity. τ is the phonon relaxation time. The mean free path between two collisions of a phonon can be defined as $L_{Ly}=\bar{\upsilon }\tau$. Thus, the thermal conductivity of the silicon can be rewritten as [30].

(4) $$\lambda =\frac{1}{3}C_{V}\bar{\upsilon }L_{Ly}$$

### 2.1. The Correction of Thermal Conductivity

The heat diffusion in silicon is dominated by phonon transports, even in the presence of large concentrations of free carrier. For further precisely modeling the thermal conductivity in the FD SOI MOSFET, the scattering of phonons and the temperature inner device should be taken into consideration because they significantly influence the heat generation and diffusion process. In Reference [23], the thermal conductivity redefined by the lattice heat capacity and the phonon scattering with complex affecting factors, including the temperature, the doping and the silicon film thickness, is expressed as

(5) $$\lambda \left(T\right)=\frac{1}{3}\sum_{j=L,T,TU}\upsilon_{j}^{2}\int_{0}^{\frac{\Theta_{D}}{T}}C_{V,j}\left(\chi_{\omega }\right)\cdot \tau_{j}\left(\chi_{\omega }\right)d\chi_{\omega }$$

where *L*,*T*,TU stand for the longitudinal, low and high frequency transverse model, respectively. $C_{V,j}$, $\upsilon_{j}$, $\Theta_{D}$ and $\tau_{j}$ the phonon-specific heat per unit volume, the phonon group velocity, the Debye temperature of solid and the phonon relaxation time, respectively. ω is the phonon angular frequency. The dimensionless phonon frequency $\chi_{\omega }$ can be defined as

(6) $$\chi_{\omega }=\frac{\hbar \omega }{2k_{B}T}$$

where *ℏ* is the Dirac constant and $k_{B}$ is the Boltzmann constant.

The phonon scattering rate becomes a significant parameter for modeling the thermal conductivity of the silicon layer in the nanoscale SOI MOSFET. It dramatically varies with the changes of the phonon scattering position in the silicon film. For a more accurate prediction of thermal conductivity in UTB-FD SOI MOSFET, the thermal conductivity is further modified by introducing the position effects terms in the silicon epitaxy film [26], which is expressed as

(7) $$\lambda \left(y,T\right)=\lambda \left(T\right)\int_{0}^{\frac{\pi }{2}}\sin^{3}\theta \left[1-\exp\left(-\frac{t_{si}}{2L_{Ly}\left(T\right)\cos\theta }\right)\times \cosh\left(\frac{t_{si}-2y}{2L_{Ly}\left(T\right)\cos\theta }\right)\right]d\theta$$

where the y axis is perpendicular to the silicon film plane. The interface between the channel and the BOX layer is at y=0, and $y=t_{si}$ stands for the top surface at the interface between the channel and the gate oxide. The θ is the angle between the y axis along the silicon thickness direction and the phonon velocity in the polar coordinates. It is an important integration parameter with a range of 0–π/2 in the thermal conductivity models. $L_{Ly}\left(T\right)$ indicates the temperature-dependent mean free path of the phonon in the silicon film.

### 2.2. Heat Capacity of Silicon Film

The lattice heat capacity is an indispensable parameter for modeling the Nano-silicon layer thermal conductivity in the MOSFETs. It will tend to be constant in a high temperature regime caused by self-heating effects, according to the Dulong-Petit law. The heat capacities of a lattice and the carriers are respectively determined by the lattice vibration and the kinetic energy of the electrons in MOSFETs. It entails that the heat capacity of the lattice is much larger than that of the electron in the device. Thus, mainly the lattice heat capacity is discussed in this work. The average energy of harmonic oscillator $k_{B}T$ can be obtained based on the principle of the equipartition of energy. Considering the quantization of the harmonic oscillator energy, the average energy can be expressed as shown in the following equation instead of $k_{B}T$.

(8) $$E\left(\omega_{i}\right.,\left.T\right)=\left(\frac{1}{2}+\bar{n}\right)\hbar \omega$$

By introducing the state density of lattice wave function expressed as

(9) $$\int_{0}^{\omega_{m}}g\left(\omega \right)d\omega =3NS$$

where 3NS is the number of lattice waves, the heat capacity at constant volume can be defined as

(10) $$C_{V}={\left(\frac{\partial U}{\partial T}\right)}_{V}=\frac{\partial }{\partial T}\int_{0}^{\omega_{m}}g\left(\omega \right)\bar{E}\left(\omega ,\left.T\right)\right.d\omega$$

where *U* is the total lattice vibration energy of silicon. Substitute Equation (8) into Equation (10), we can get

(11) $$C_{V}=\int_{0}^{\omega_{D}}k_{B}{\left(\frac{\hbar \omega }{k_{B}T}\right)}^{2}\frac{\frac{\hbar \omega }{{e^{k_{B}}}^{T}}}{\left(e^{\frac{\hbar \omega }{k_{B}T}}-1\right)}g\left(\omega \right)d\omega$$

where $\omega_{D}$ is the Debye frequency. So far, the problem of solving the solid heat capacity is transformed to the solution of the state density of the lattice wave function. However, it is too difficult to solve the state density of the lattice wave function for a specific crystal like silicon. Thus, the Debye model was introduced to simplify the solving process and Equation (11) is rewritten as

(12) $$C_{V}=\frac{3V}{2\pi^{2}v_{p}^{3}}\int_{0}^{\frac{\Theta_{D}}{T}}\frac{k_{B}^{4}T^{3}}{\hbar^{3}}\chi_{\omega }^{4}\frac{e^{\chi \omega }}{{\left(e^{\chi }\omega -1\right)}^{2}}d\chi_{\omega }=9Nk_{B}{\left(\frac{T}{\Theta_{D}}\right)}^{3}\int_{0}^{\frac{\Theta_{D}}{T}}\frac{\chi_{\omega }^{4}e^{\chi \omega }}{{\left(e^{\chi }\omega -1\right)}^{2}}d\chi_{\omega }$$

where $\upsilon_{p}$ is acoustical lattice wave phase velocity.

### 2.3. Analysis of Phonon Scattering

The phonon scattering plays a significant role in the silicon layer of the nanoscale SOI MOSFET. With the thickness of the silicon film decreasing, the effects on phonon scattering of boundary reflection and bound electrons should be considered, especially for ultra-thin body SOI devices. The phonon scattering rate in Asheghi’s work can be improved by introducing these extra scattering mechanisms.The differences between the two models presented in Asheghi’s and our previous work [27] are indicated in Equation (13).

(13) $$\tau^{-1}=\tau_{p,Nb}^{-1}+\tau_{impurity}^{-1}+\tau_{free-elec}^{-1}+\tau_{bound-elec}^{-1}+\tau_{Ly}^{-1}$$

where $\tau_{p,Nb}^{-1}$ indicates the phonon scattering rate in pure bulk silicon material, which is summarized in previous works with empirical coefficients [31,32].

$\tau_{impurity}^{-1}$ is the increased scattering rate caused by dopant atoms in the silicon film. It is defined as

(14) $$\tau_{impurity}^{-1}=\left(A_{\delta M}+A_{\delta R}+A_{x}\right)\omega^{4}$$

where $A_{x}\omega^{4}$ is the correction term. The difference of the mass between the silicon atoms and the dopant atoms results in the variability of the scattering phonon velocity seriously influencing the phonon scattering rate. $A_{\delta M}\omega^{4}$ is used to describe the impact of the dopant mass on the phonon scattering rate and can be given by

(15) $$A_{\delta M}=\frac{nV^{2}}{4\pi \upsilon_{S}^{3}}{\left(\frac{\delta M}{M}\right)}^{2}$$

where, *n* is the concentration of the point imperfections per volume, *M* and *V* are the mass and crystal volume of the silicon atom, respectively. δM is the mass difference between the dopant and the silicon atom. $A_{\delta R}\omega^{4}$ which is used to indicate the impact of the strain in the lattice on the phonon scattering after doping can be defined as

(16) $$A_{\delta R}=\frac{2nV^{2}}{\pi \upsilon_{S}^{3}}Q_{0}^{2}\gamma^{2}{\left(\frac{\delta R}{R}\right)}^{2}$$

The parameter $Q_{0}$ depends on how the nearest and further-out linkages combined in the scattering matrix. The Gruneisen constant γ is obtained from thermal expansion data. The radii of the normal ions and the difference between the radii of the foreign and normal ions are *R* and δR respectively.

$\tau_{free-elec}^{-1}$ and $\tau_{bound-elec}^{-1}$ reveal the impacts of the electron on the thermal conductivity. Considering the influence of doping concentration on the electron state in the phonon scattering process, the donor impurity concentration should be taken into account to get a more accurate estimate of the thermal conductivity. The boundary concentration introduced for defining the electron state in silicon is expressed as

(17) $$n_{t,Si}={\left(\frac{m_{e}q_{e}^{2}}{16\pi \varepsilon_{Si}\hbar^{2}}\right)}^{3}$$

where $m_{e}$ is the mass of the electron. $q_{e}$ is the electron charge. $\varepsilon_{Si}$ is the relative dielectric constant of silicon. The doped silicon can be regarded as non-metal material, including the insulation and transition states, when the doping concentration is lower than the boundary concentration.

As for the insulation state of the low-doped silicon, the phonon scattering due to the bound electron could be simplified to the collisions between unit cell and phonon, which results in the shear deformation taking place in the unit cell and the energy achieved by collisions would transfer to lattice potential energy. The relaxation time was expressed as

(18) $$\begin{array}{c} \tau_{bound-elec}^{-1}=\frac{\omega^{4}{\left(0.33\Xi_{u,elec}\right)}^{4}}{10\pi \rho^{2}\upsilon_{S}^{2}}f^{2}\left(\frac{\omega }{\nu }\right)\left\{\upsilon_{L}^{-5}f^{2}\left(\frac{\omega }{\upsilon_{T}}\right)+\upsilon_{L}^{-5}f^{2}\left(\frac{\omega }{\upsilon_{L}}\right)\right\}\\ \times 3w_{ave}\frac{2\Delta^{2}}{{\left(\Delta^{2}-\hbar^{2}\omega^{2}\right)}^{2}}{\left[2\left(n_{0}+n_{1}\right)+n_{1}\left(1+\frac{\Delta^{2}}{\hbar^{2}\omega^{2}}\right)\right]}^{2} \end{array}$$

(19) $$f\left(q\right)={\left[1+\frac{{\left(a_{B}^{\prime }\right)}^{2}q^{2}}{4}\right]}^{-2}$$

where, ρ is the crystal density. $\Xi_{u,elec}$ is the shear deformation potential constant. $\upsilon_{L}$ and $\upsilon_{T}$ are the velocities of longitudinal and transverse elastic waves, respectively. Δ=13meV is the difference value of states between a single and a doublet. $n_{0}$ and $n_{1}$ are the density of the electron in states of singlet and doublet. $w_{ave}$ is the average weighting coefficient for the change between before and after the collision of phonon. *q* is the phonon wave vector. $a_{B}^{\prime }$ is the Bohr radius of silicon [27].

The effects of bound electrons and free electrons must be considered simultaneously for the phonon scattering rate in the transition material. The electron-phonon scattering rate is defined using a correction term, based on the relaxation time of free electron scattering.

(20) $$\tau_{trans-elec}^{-1}=\tau_{free-elec}^{-1}+\tau_{bound-elec}^{-1}=\tau_{0}\times K$$

(21) $$\tau_{0}=\frac{{\left(m_{e}E_{D}\right)}^{2}k_{B}T}{2\pi \rho \hbar^{4}\upsilon^{2}}$$

(22) $$K=\ln\left[\frac{1+\exp\left(\zeta -\zeta_{0}\frac{\chi_{\omega }^{2}}{16}+\frac{\chi_{\omega }}{2}\right)}{1+\exp\left(\zeta -\zeta_{0}\frac{\chi_{\omega }^{2}}{16}-\frac{\chi_{\omega }}{2}\right)}\right]$$

(23) $$\zeta =\frac{m_{e}\upsilon^{2}}{2k_{B}T}$$

where $E_{D}$ is deformation potential. $\tau_{0}$ is the expression of the effect of a single free electron on the phonon scattering rate. *K* is the correction term. $\zeta_{0}$ is the energy gap between Fermi level and the conduction band.

The impact of the free electron on the phonon scattering rate is considered in the metal state silicon heavily doped beyond boundary concentration and it is expressed as

(24) $$\tau_{free-elec}^{-1}=\frac{{\left(m_{e}E_{D}\right)}^{2}k_{B}T}{2\pi \rho \hbar^{4}\upsilon^{2}}\times \chi_{\omega }$$

$\tau_{Ly}^{-1}$ represents the part of phonon scattering rate induced by boundary reflection on the phonon scattering. It is determined by the aggregate influence of the former four parts of Equation (13) with the boundary scattering reduction factor *F* which is influenced by the thickness of silicon film $t_{si}$, the mean free path of phonon, the reduced thickness δ, the auxiliary parameter $t_{0}$ and the specular reflection coefficient *p* used to define phonon scatters specularly, or diffusively at the interface [33].

(25) $$\tau_{Ly}^{-1}=\left(\tau_{p,Nb}^{-1}+\tau_{impurity}^{-1}+\tau_{free-elec}^{-1}+\tau_{bound-elec}^{-1}\right)\cdot F\left(\frac{t_{si}}{L_{Ly}},p\right)$$

(26) $$F\left(\delta ,\eta \right)=1-\frac{3\left(1-P\right)}{2\delta }\int_{1}^{\infty }\left(\frac{1}{{t_{0}}^{3}}-\frac{1}{{t_{0}}^{5}}\right)\times \frac{1-\exp\left(-\delta t_{0}\right)}{1-p\exp\left(-\delta t_{0}\right)}dt_{0}$$

(27) $$\delta =\frac{t_{si}}{L_{Ly}}$$

Here, *p* can be estimated from the characteristic dimension of surface roughness η, the incident wave-vector *q* and the incident angle $\theta_{0}$ by [34]

(28) $$p\left(q,\eta \right)=\exp\left\{-{\left(2\eta q\cos\left(\theta_{0}\right)\right)}^{2}\right\}$$

It is also customary to determine the actual specularity for each phonon using the expression $p\left(q,\eta \right)=\exp\left\{-{\left(2\eta q\right)}^{2}\right\}$, which is strictly valid only for phonons whose path before striking the surface is normal to the surface [35].

## 3. Results and Discussion

**Figure 1:**
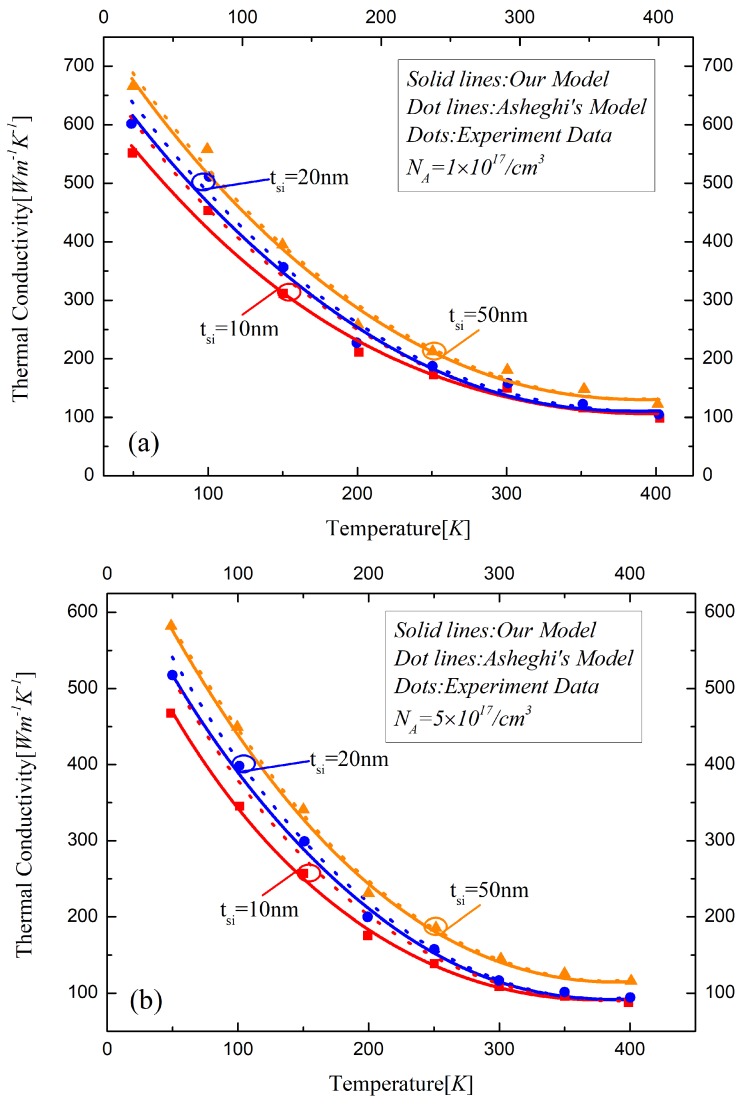

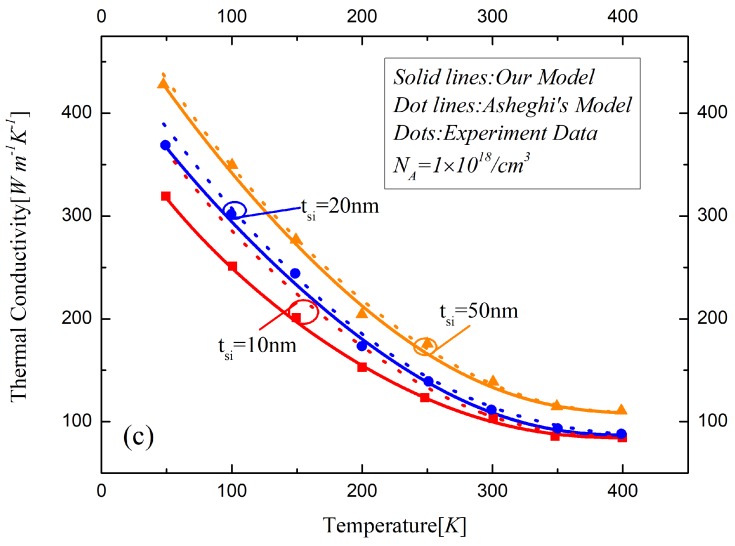
The thermal conductivity of the ultra-thin silicon film material with different thickness and various doping concentration considering the influence of temperature (**a**) $N_{A}=1\times 10^{17}$/cm^3^, (**b**) $N_{A}=5\times 10^{17}/cm^{3}$, (**c**) $N_{A}=1\times 10^{18}/cm^{3}$.

Figure 1 shows the thermal conductivity for the phosphorus doped silicon layer of SOI MOSFETs predicted by the analytical model in this work and Asheghi’s model. It can be seen that the models match well with the experimental data by the Transient Plane Source method [36]. The difference between these two works becomes larger with the thickness of silicon decreasing from 50 nm to 10 nm because of the increased phonon boundary effects in the nanoscale silicon film. It shows that the heat diffusion was suppressed by the increase in temperature, and the thermal conductivity remains at a high temperature, revealed by the Dulong-Petit law. The reflection of phonons at the boundary determines the thermal characteristic when the thickness of the silicon layer between the gate and the buried oxide decreases to sub-20 nm. The boundary scattering could be modeled by a rough surface reflection process which is irrelevant to the thickness of silicon at a high temperature, entailing the same constant values of the thermal conductivity of 10 nm and 20 nm silicon film. However, the heat loss process in the 50 nm-thickness silicon film material is mainly influenced by the other factors, resulting in the larger difference of thermal conductivity in a high temperature regime because the boundary effects are inversely proportional to the thickness.

**Figure 2:**
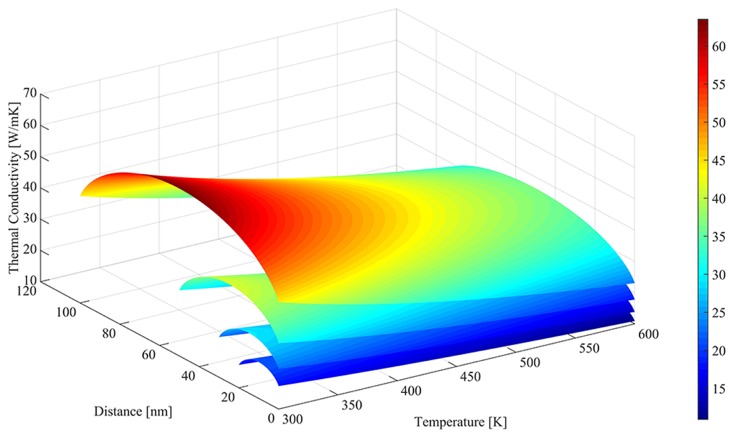
Depth and temperature dependence of the thermal conductivity of the ultra-thin body fully depleted (FD) silicon-on-insulator (SOI) metal-oxide-semiconductor field-effect transistor (MOSFET) for different silicon film thicknesses of 20 nm, 40 nm, 50 nm and 100 nm.

**Figure 3:**
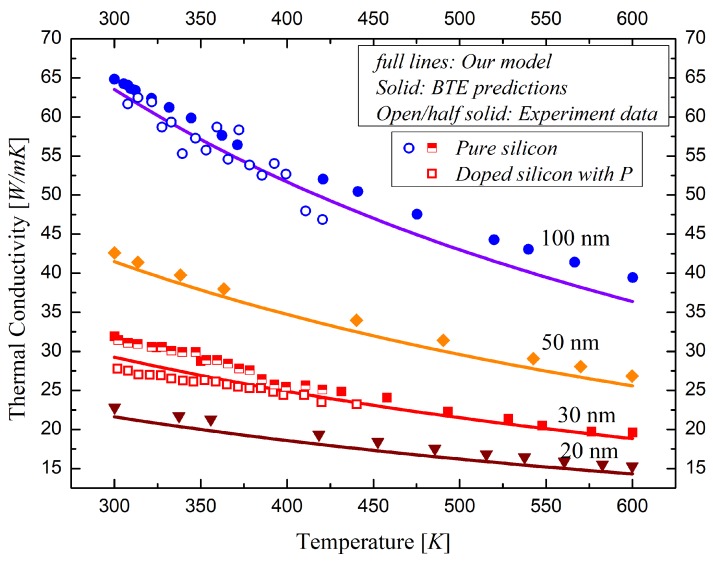
The temperature dependence of the thermal conductivity for FD SOI device compared with the BTEpredictions and the experimental data in previous works [19].

Based on the analysis of the thermal characteristic of silicon film, the reduction factor is adopted to model the temperature and the depth dependence of thermal conductivity, as shown in Figure 2. It shows that the thermal conductivity of silicon film is reduced dramatically in UTB FD SOI devices and further decreased approaching the Si/BOX and Si/Gate-oxide interfaces. The average thermal conduction versus temperature for different thicknesses of silicon layers is given in Figure 3 by comparing with the BTE predictions and the experiment data in previous works. The experimental data on pure silicon is very close to the BTE predictions. The thermal conductivity calculated by our model is a bit lower than that of pure silicon and the BTE data and close to that of phosphorus doped silicon film because of the impurity scattering.

**Figure 4:**
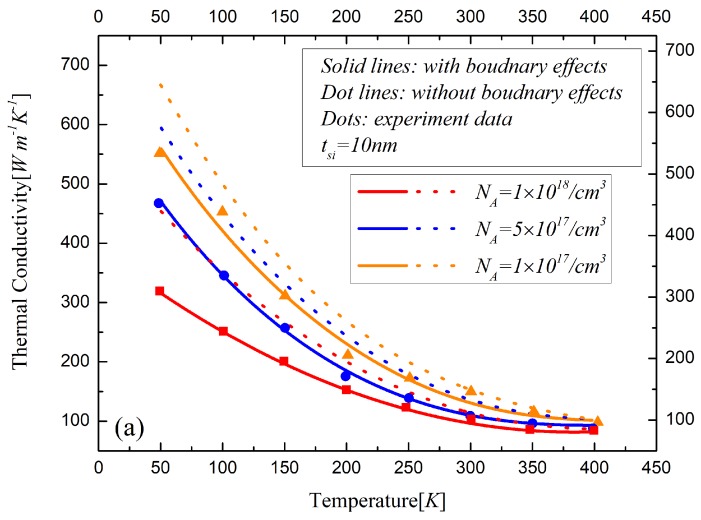

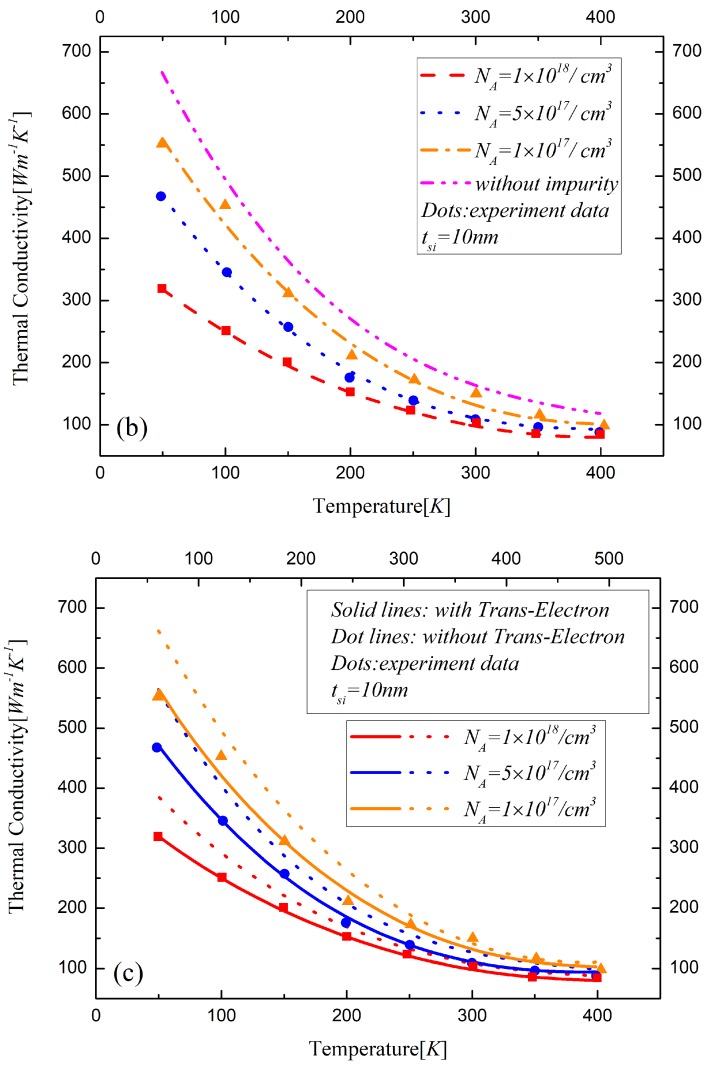
The influences of (**a**) boundary scattering effects, (**b**) impurity, (**c**) free and bound electron on the thermal conductivity of the 10 nm-thickness silicon film material in ultra-thin body FD-SOI MOSFET.

**Figure 5:**
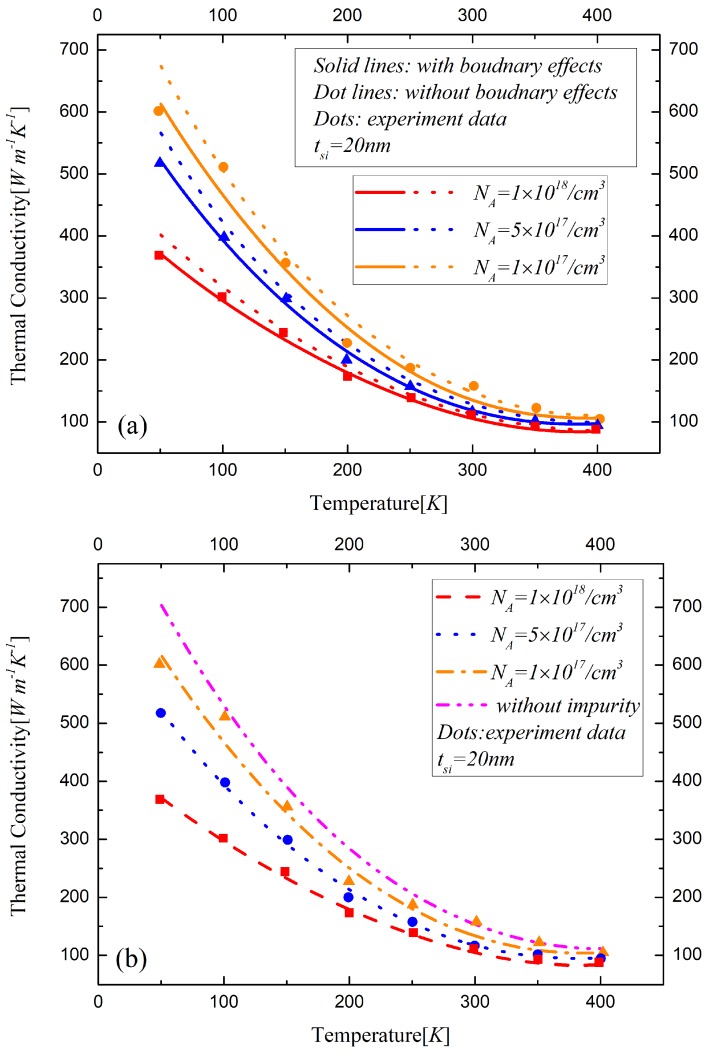

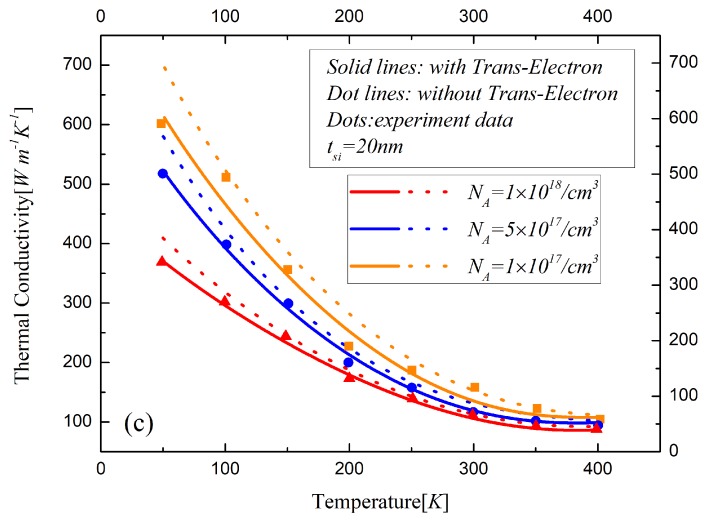
The influences of (**a**) boundary scattering effects, (**b**) impurity, (**c**) free and bound electron on the thermal conductivity of the 20 nm-thickness silicon film material in ultra-thin body FD-SOI MOSFET.

**Figure 6:**
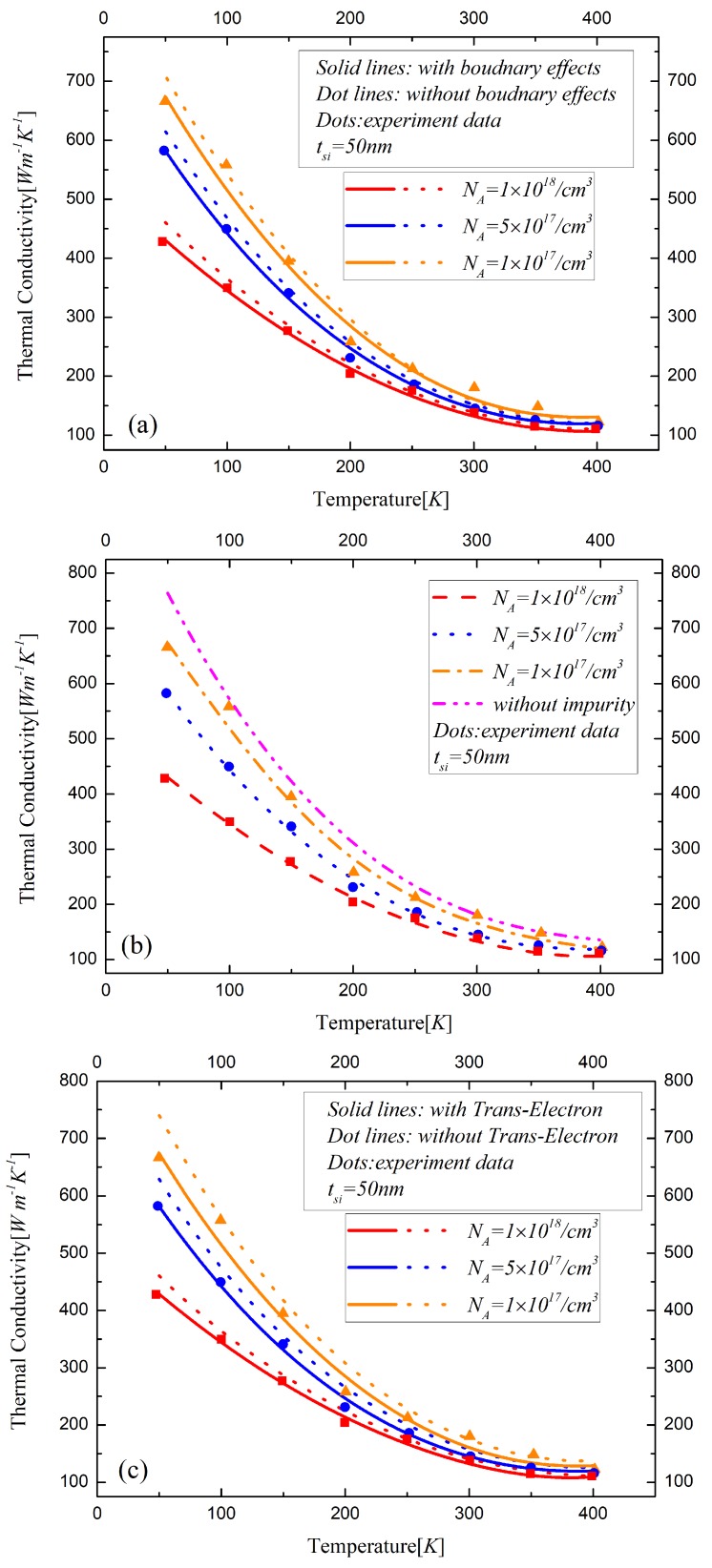
The influences of (**a**) boundary scattering effects, (**b**) impurity, (**c**) free and bound electron on the thermal conductivity of the 50 nm-thickness silicon film material in ultra-thin body FD-SOI MOSFET.

The electron states in the silicon layer determined by the boundary concentration described in Equation (17) are used to discuss the effects of the free and bound electrons on the phonon scattering rate. It indicates that the silicon layer with a high doping concentration of $10^{18}/cm^{3}$ is in a metallic state where the electron can be considered in a free state. When the doping concentration is lower than $2.5\times 10^{17}/cm^{3}$, the silicon film can be treated as in insulation state because of the bound electron. The states of the silicon film will entail different phonon scattering rates, resulting in changes to the thermal conductivity. Between the insulation states and metallic states, there are transition states in which the bound electrons are activated to some extent. Thus, the silicon films with different doping concentrations of $N_{A}=1\times 10^{17}/cm^{3}$, $N_{A}=5\times 10^{17}/cm^{3}$ and $N_{A}=1\times 10^{18}/cm^{3}$, respectively, are listed in the analysis of thermal conductivity. The influences of various phonon scattering effects on the thermal conductivity are studied for UTB FD SOI MOSFETs with a 25 nm channel length and 10 nm, 20 nm and 50 nm doped silicon layers, as shown in Figure 4, Figure 5 and Figure 6, respectively.

The boundary effects on the thermal conductivity are demonstrated in Figure 4a, Figure 5a and Figure 6a, which actually indicate the difference between our model and Asheghi’s model. The influences of impurity on thermal conductivity are analyzed in Figure 4b, Figure 5b and Figure 6b. The surrounding atoms are displaced by the insertion of the impurities, which will induce the lattice strain. The velocities of the phonons, which are changed by the variations along the interatomic distance, will lead to direction changes and phonon scattering events [25]. The relative displacements of the neighboring atoms increase the relaxation time resulting in the decrease of thermal conductivity. Figure 4c, Figure 5c and Figure 6c show the difference of the thermal conductivity models with and without phonon-electron in silicon material with different states. The bound electrons increase due to low donor impurities. As for the high concentration doped silicon layers, the electrons are free to move in the conduction band. The observed reduction in the lattice thermal conductivity with high doping is due to the phonon scattering on free carriers. The thermal conductivity decreases with the temperature and the doping concentration increasing in the device. With the thickness of the silicon layer decreasing, the influences of various factors of the phonon scattering on the thermal conductivity significantly increase, especially the impact of the boundary effects, which are remarkable in sub-20 nm regime.

In order to further reveal the dominant degree of different scattering mechanisms, the coefficient of determination is adopted to quantify the influences of the various scattering mechanisms and to analyze the merits of the thermal conductivity model. Because the proposed models are highly nonlinear, the coefficient of determination for the linear regression makes no sense for quantifying the reliability of the different models with a high degree. A nonlinear coefficient of determination ($R^{2}$) was adopted for the following analysis, the value of which is limited in the range of 0∼1. It can be defined as

(29) $$R^{2}\left(\lambda_{ex},\lambda_{m}\right)=1-\sqrt{\frac{\sum_{i=1}^{N}{\left(\lambda_{ex,i}-\lambda_{m,i}\right)}^{2}}{\sum_{i=1}^{N}\lambda_{ex,i}^{2}}}$$

where the $\lambda_{ex}$ and the $\lambda_{m}$ are the experimental data and the model calculation results of the thermal conductivity. *N* is the number of the experiment data.

**Figure 7:**
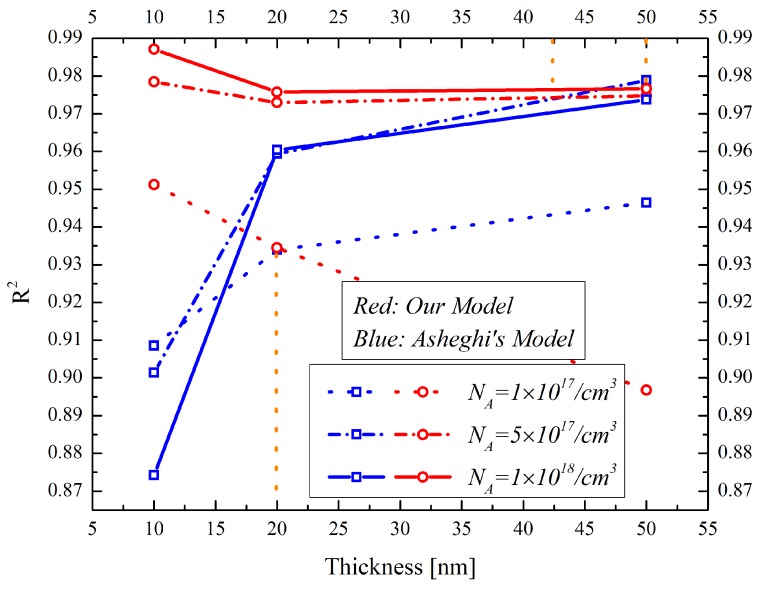
The coefficients of determination in the nonlinear regression analysis of the thermal conductivity models with different doping concentration discussed in this paper.

The coefficients of determination are calculated for the reliability comparison between both models. Figure 7 shows the coefficients of determination in the nonlinear regression analysis of the thermal conductivity models with different doping concentrations. The coefficients of determination of our model increase as the silicon layer thicknesses decrease and the doping concentrations increase. This is because the boundary scattering can be enhanced by decreasing the silicon layer thickness and by increasing the doping concentration. However, the coefficients of determination of Asheghi’s model decrease as the silicon thickness decreases and as the doping concentration increases. This indicates that the thermal conductivity predictive power of the Asheghi’s model degrades in the sub-10-nm thicknesses and in the heavily doped silicon layers. It can be concluded that with the thickness of the silicon layer decreasing down to sub-20 nm, the thermal conductivity prediction of our model for the $10^{18}/cm^{3}$ doped silicon layer is more precise than that of Asheghi’s model. The values of the coefficients of determination could reach as large as 0.95 in the sub-10 nm silicon thickness regime. There is a critical silicon layer thickness point that can be used to distinguish the superiority of the two models. The thicknesses are 20 nm, 42 nm and 50 nm for the $N_{A}=1\times 10^{17}/cm^{3}$, $N_{A}=5\times 10^{17}/cm^{3}$ and $N_{A}=1\times 10^{18}/cm^{3}$ doped silicon layer, respectively. It can be seen that the critical silicon layer thickness increases as the $N_{A}$ increases because of the increased boundary reflection scattering.

**Figure 8:**
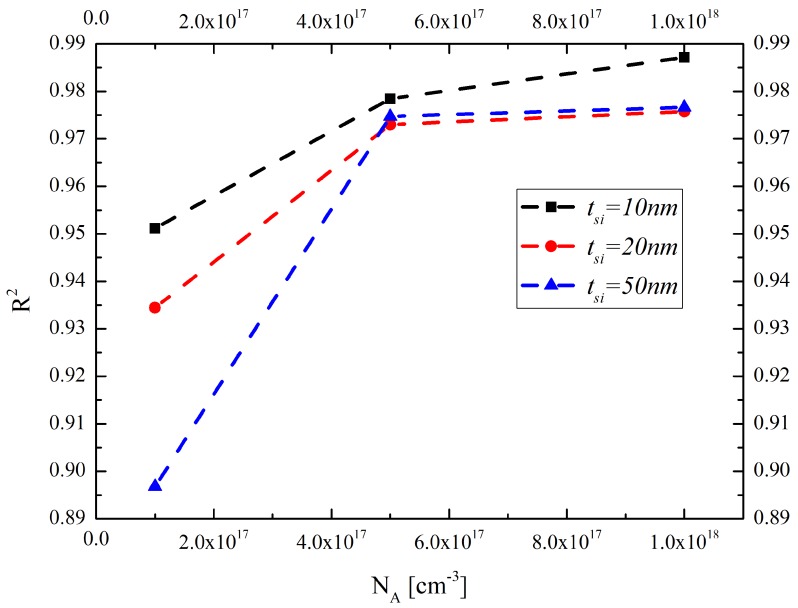
The influences of the doping concentration and the silicon layer thickness on fitting goodness of the thermal conductivity model considering the boundary scattering effect.

Figure 8 shows the influences of the doping concentration and the silicon layer thickness on the fitting goodness of the thermal conductivity model, considering the boundary scattering effect. It indicates that the model will possess a high reliability for thermal conductivity prediction in sub-10 nm and heavy doping concentration silicon layers due to the fierce boundary reflection scattering.

**Figure 9:**
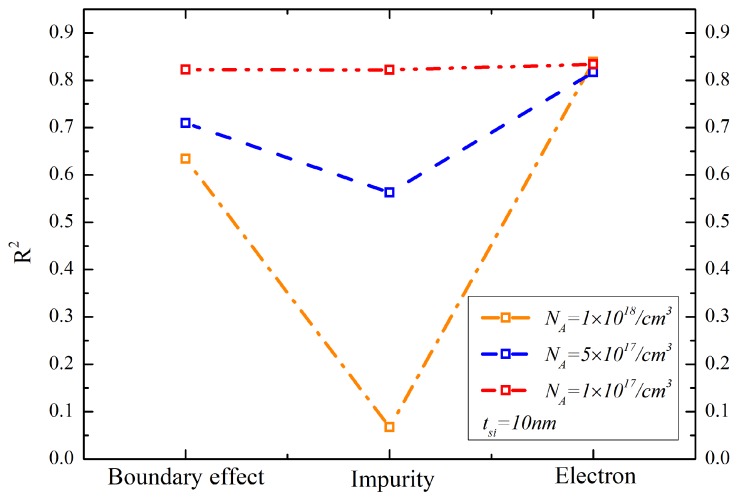
The reliability of the model influenced by the boundary, impurity-phonon and electron-phonon scattering mechanisms in the 10-nm silicon layer.

The reliability of the thermal conductivity model determined by the boundary, impurity-phonon and electron-phonon scattering mechanisms in the 10-nm silicon layer is quantified in Figure 9. The absence of a scattering mechanism in the model will make the coefficients of determination less than 0.85 in the $1\times 10^{17}/cm^{3}$ doped silicon layer. Moreover, when the impurity-phonon scattering is ignored in the $1\times 10^{18}/cm^{3}$ doped 10-nm silicon layer, the coefficient of determination is less than 0.1. This means that the model makes no sense for thermal conductivity prediction. The higher doping concentration entails the stronger impact of the boundary scattering and the impurity-phonons on the thermal conductivity. It indicates that all of the scattering mechanisms play an important role in thermal conductivity prediction in the nanoscale SOI MOSFETs. The phonon-boundary scattering dominates the phonon-impurity scattering at low temperatures. Phonon-boundary scattering also contributes significantly to the reduction in the thermal conductivity of silicon layers regardless of the level of impurity concentration.

## 4. Conclusions

In this paper, analytical modeling of thermal conductivity based on the phonon scattering mechanism is studied for nanoscale UTB-FD SOI MOSFETs with different parameters including temperature, depth, thickness and doping concentration. When compared with the BTE predictions and the experimental data from previous works, the results calculated are close to that of pure silicon and the BTE data and that of phosphorus doped silicon layers and thus the model can be verified to some extent. When compared with Asheghi’s model, the predictive power of our model is enhanced for devices with sub-10-nm thickness and a heavily doped silicon layer, while considering the boundary scattering contribution, and for devices with sub-20-nm thickness and a heavily doped silicon layer due to the dramatic enhanced phonon boundary reflection mechanism. All of the scattering mechanisms, including the phonon-impurity, the phonon-electron and the boundary scattering, play an important role in the thermal conductivity prediction.
